# Prevalence and risk of burnout among HIV service providers in South Africa and Zambia: findings from the HPTN 071 (PopART) trial

**DOI:** 10.1186/s12960-024-00934-9

**Published:** 2024-07-08

**Authors:** Mara C. Steinhaus, Tamaryn J. Nicholson, Triantafyllos Pliakas, Abigail Harper, Pamela Lilleston, Tila Mainga, Deborah Milimo, Karen Jennings, Nelis Grobbelaar, Francoise Louis, Handri Liebenberg, Richard J. Hayes, Sarah Fidler, Helen Ayles, Peter Bock, Graeme Hoddinott, James R. Hargreaves, Virginia Bond, Anne L. Stangl, the HPTN 071 (PopART) study team

**Affiliations:** 1 International Center for Research On Women, Washington, DC USA; 2 WomenStrong International, Washington, DC USA; 3 Desmond Tutu TB Centre, Department of Paediatrics and Child Health, Faculty of Medicine and Health Sciences, Stellenbosch University, Stellenbosch, South Africa; 4 Department of Global Health and Development, London School of Hygiene and Tropical Medicine, London, UK; 5 Impact Epilysis, Thessaloniki, Greece; 6 GSK Vaccines, Wavre, Belgium; 7 Zambart, School of Public Health, University of Zambia, Lusaka, Zambia; 8 Department of Health, City of Cape Town, HIV/AIDS, STIs, and TB, Cape Town, South Africa; 9 Anova Health, Johannesburg, South Africa; 10 Kheth’Impilo, Cape Town, South Africa; 11 Western Cape, Department of Health, Cape Town, South Africa; 12 Department of Infectious Disease Epidemiology, Faculty of Epidemiology and Population Health London School of Hygiene and Tropical Medicine, London, UK; 13 Department of Medicine, Imperial College London, London, UK; 14 Department of Clinical Research, Faculty of Infectious and Tropical Diseases, London School of Hygiene and Tropical Medicine, London, UK; 15 Department of Public Health, Environments and Society, Faculty of Public Health and Policy, London School of Hygiene and Tropical Medicine, London, UK; 16 Department of International Health, Johns Hopkins Bloomberg School of Public Health, Baltimore, MD USA

**Keywords:** Maslach Burnout Inventory, HIV, Facility-based health workers, Community-based health workers, Emotional exhaustion, Stigma

## Abstract

**Background:**

In the high disease burden and resource-constrained contexts of sub-Saharan Africa (SSA), health workers experience a range of psychosocial stressors that leave them vulnerable to developing burnout, which can reduce service quality and negatively impact their own health and wellbeing. As universal testing and treatment (UTT) for HIV scales up across SSA, we sought to understand the implications of this human resource-intensive approach to HIV prevention to inform decision-making about health workforce staffing and support needs.

**Methods:**

Using the Maslach Burnout Inventory-Human Services Survey (MBI-HSS), we assessed the prevalence of three domains of burnout—emotional exhaustion, depersonalization, and personal accomplishment—among three cadres of health workers delivering health services in areas receiving a UTT intervention in Zambia and South Africa. These cadres included health facility workers (*n* = 478), community health workers (*n* = 159), and a study-specific cadre of community HIV care providers (*n* = 529). We used linear regression to assess risk factors associated with emotional exhaustion, the only domain with sufficient variation in our sample.

**Results:**

The MBI-HSS was completed by 1499/2153 eligible participants (69.6% response rate). Less than 1% of health workers met Maslach’s definition for burnout. All groups of health workers reported lower levels of emotional exhaustion than found in previous studies of this type (mean score scores ranged from 10.7 to 15.4 out of 54 across health cadres). Higher emotional exhaustion was associated with higher educational attainment (*β*adj = 2.24, 95% CI 0.76 to 3.72), greater years providing HIV services (*β*adj = 0.20, 95% CI 0.03 to 0.36), and testing negative for HIV at last HIV test (*β*adj = − 3.88 − 95% CI 5.69 to − 2.07). Working as a CHW was significantly associated with lower emotional exhaustion (*β*adj = − 2.52, 95% CI − 4.69 to − 0.35). Among all health workers, irrespective of HIV status, witnessing stigmatizing behaviors towards people living with HIV among their co-workers was associated with significantly increased emotional exhaustion (*β*adj = 3.38, 95% CI 1.99 to 4.76).

**Conclusions:**

The low level of burnout detected among health workers is reassuring. However, it remains important to assess how UTT may affect levels of emotional exhaustion among health workers over time, particularly in the context of emerging global pandemics, as burnout may impact the quality of HIV services they provide and their own mental health and wellbeing. Interventions to reduce HIV stigma in health facilities may protect against emotional exhaustion among health workers, as well as interventions to increase mindfulness and resilience among health workers at risk of burnout.

*Trial registration* ClinicalTrials.gov number: NCT01900977.

**Supplementary Information:**

The online version contains supplementary material available at 10.1186/s12960-024-00934-9.

## Background

As of 2020, there were an estimated 7.8 million people living with HIV (PLHIV) in South Africa and 1.5 million in Zambia, 79% and 81% of whom were taking antiretroviral therapy (ART), respectively [1, 2]. In line with the World Health Organization’s 2016 recommendations to initiate treatment for all PLHIV, regardless of CD4 count (the number of CD4 cells in a person’s blood), both Zambia and South Africa implemented ART policies in 2016, adopting a universal treatment approach [3, 4]. A universal treatment approach requires increased staffing, not only to test and initiate new patients into care, but also to retain them in care and support adherence to ART. One study conducted in 2009 indicated that implementation of universal treatment in high prevalence sub-Saharan African (SSA) settings would require over 17,000 additional health workers and that to initiate universal access to treatment at CD4 cell counts of ≤ 350 cells/μl, South Africa alone would require a minimum of 6300 additional health workers [5].

In addition to an expanded health workforce needed to support UTT, many qualitative studies report that health workers in SSA already see their current care targets as unattainable, with some health workers compensating by using time outside of working hours, as well as personal resources, to deliver care to patients in need [6–10]. Such conditions may increase the risk of burnout among health workers in SSA as UTT is rolled out more broadly and highlight the need to track burnout closely among the health workforce.

Occupational burnout describes a state of mental exhaustion, demotivation, and cynicism in the workplace, which can negatively impact organizational outcomes [11]. In the high-disease-burden and resource-constrained contexts of SSA, health workers experience a range of psychosocial stressors that leave them vulnerable to developing burnout, in many cases reducing service-quality as well as negatively impacting on their own health and wellbeing [12]. Health workers also face daily interpersonal, organizational, and structural threats to their mental wellbeing [9, 10]. Caring for PLHIV may expose health workers to secondary, or associative, stigma simply through providing services to members of a stigmatized group [13–15]. Such stigma has been reported previously by community health workers employed to conduct follow-up and adherence monitoring in Zambia [14, 16]. In the context of rapid expansion of HIV testing and treatment services in high burden low resource settings, it is imperative to gain a better understanding of health worker occupational burnout.

### Assessing burnout

The Maslach Burnout Inventory-Human Services Survey (MBI-HSS) is a measure of burnout which is defined as “a psychological syndrome emerging as a prolonged response to chronic interpersonal stressors on the job” [17]. The measure comprised three factors or dimensions constituting burnout, these are: emotional exhaustion, depersonalization, and low personal accomplishment [18]. The measure was initially developed and validated in high income contexts, but its use in a broader range of low- and middle-income contexts has become more frequent over the last decade [18].

In SSA, a recent systematic review found that burnout has been studied mostly among nurses, physicians, medical and nursing students, and combined populations of healthcare providers, with nurses reporting the highest levels of burnout across health worker cadres in the region [19]. Less is known about burnout among community health workers. One study in South Africa, reported moderate to high levels of stress that could lead to burnout among volunteer caregivers [20]. As community health workers, both paid and unpaid, are relied on in SSA to provide HIV testing, care, and support in community settings [21], it will be critical to better understand burnout among this health worker cadre to inform policy decisions about the health workforce in the region.

Evidence suggests that sociodemographic characteristics including sex, age, marital status, and level of education are associated with burnout, and that these associations differ across the three dimensions of burnout [20, 22–25]. Meta-analyses suggest that women score higher on the emotional exhaustion subscale, whereas men are more likely to score higher on the depersonalization subscale, reflecting a distant or indifferent attitude towards work [22].

Occupational risk factors of burnout among healthcare workers in SSA include work environments, interpersonal and professional conflicts, low social support, and emotional distress [19]*.* Results regarding the relationship between years of practice and emotional exhaustion and depersonalization are mixed [18], possibly because of survival bias, where providers experiencing burnout leave their occupation. Few studies have explored burnout among different types of health workers (e.g., community health workers versus facility-based) or the impact of health workers’ HIV status and perceptions of co-workers’ HIV stigmatizing behaviors on their level of occupational burnout.

In this study, we aimed to: (a) describe the prevalence of three domains of burnout—emotional exhaustion, depersonalization, and personal accomplishment—among different types of health workers delivering HIV services in the context of a large universal testing and treatment trial in Zambia and South Africa, and (b) assess the association between emotional exhaustion and sociodemographic and occupational risk factors, including HIV status and perceptions of the stigmatizing behaviors of co-workers.

## Methods

### Population

**Fig. 1:**
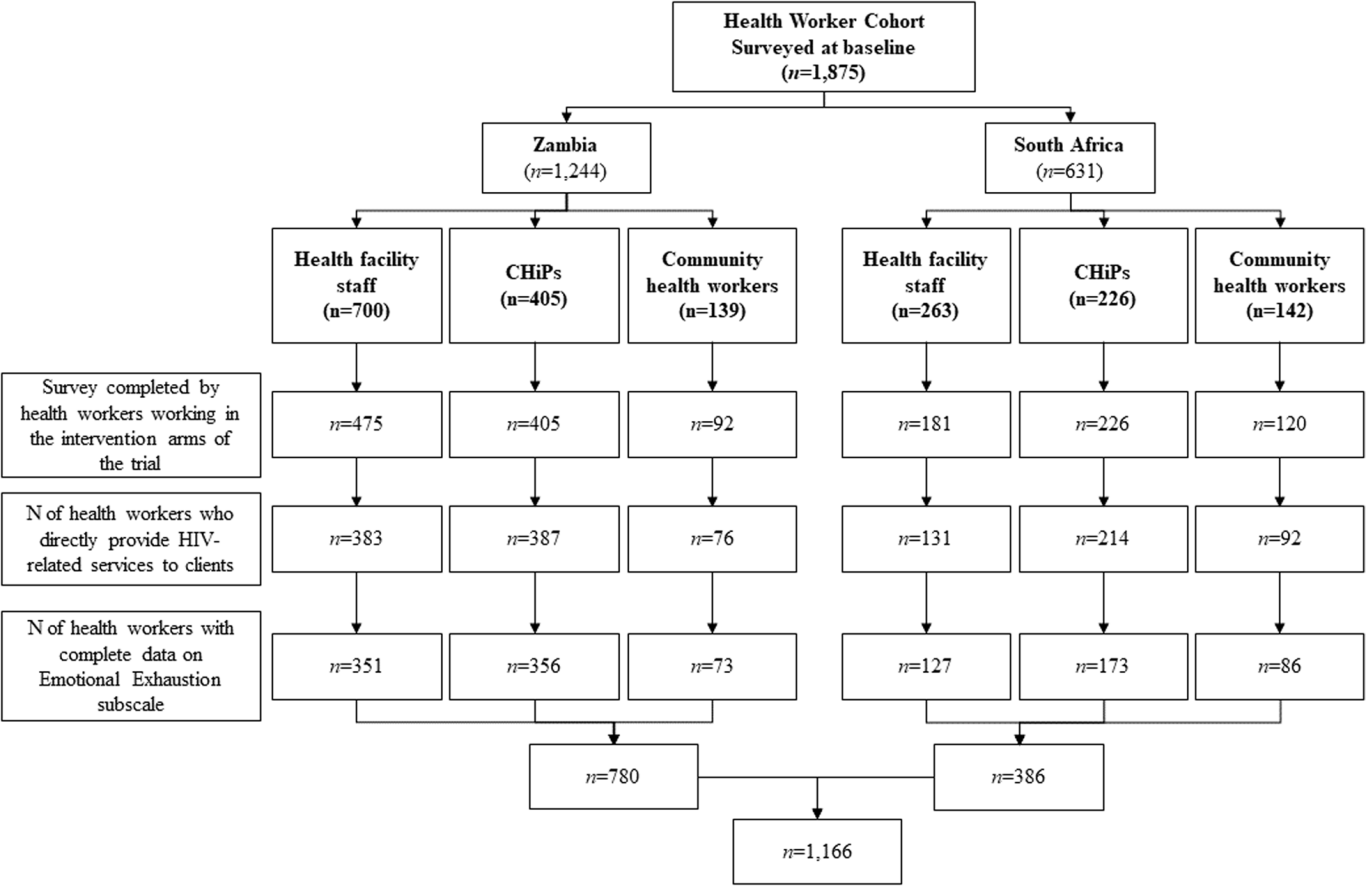
﻿Participant flow diagram

This analysis used baseline data collected between July 2014 and May 2015 as part of the first recruitment round of the HPTN 071 (PopART) stigma ancillary study among health workers [26]. Of 2833 eligible health workers across the three study arms, 1875 responded to the baseline survey, including the MBI-HSS, for an overall response rate of 66.2% [27]. For the present analysis, we only included data from the two intervention arms. Thus, our response rate was slightly higher, as 1499 out of 2153 eligible participants completed the MBI-HSS (69.6%) (Fig. 1). Quantitative surveys were conducted among three cadres of health workers—community HIV care providers (CHiPs; *n* = 631), health facility staff (HFS; *n* = 963), and community health workers (CHWs; *n* = 281) [26]. CHiPs were recruited as trial staff to provide home-based HIV testing and referral and operated in two out of the three trial arms, excluding the control arm [28]. To meet the inclusion criteria for the study, health workers were required to be at least 18 years of age and willing to provide informed consent for participation. Data were captured using electronic capture devices that allowed participants to respond directly to questions without disclosing their responses to interviewers. For the purposes of this analysis, we restricted our sample to health workers who self-reported providing HIV services at the time of the interview (*n* = 1557), who were working in the intervention arms of the trial only (*n* = 1284), and who answered all the questions for the Emotional Exhaustion subscale (*n* = 1166). Recruitment occurred between 8 to 18 months after commencement of the trial, depending on the study community. The participant flow diagram showing the final sample size is presented in Fig. 1.

Required experience and conditions of employment of the three cadres of health workers employed in this study varied in ways which are likely to impact their levels of burnout. CHiPs in Zambia were required to have previous training in HIV counseling and testing, while CHiPs in South Africa were trained after recruitment. HFS worked in 14 health facilities in South Africa and Zambia (6 in South Africa and 8 in Zambia). CHWs were existing cadres of health workers in both Zambia and South Africa. Some were formally employed and paid by non-governmental organizations (NGOs), while others were unpaid volunteers. In Zambia, the volunteer CHWs were affiliated with clinics operated by the Ministry of Health, while in South Africa, the CHWs were managed by NGOs that held tenders to provide ancillary support to the Department of Health. Historically, in both countries, these CHWs had often been in a palliative care role before ART was available. Over time, their role in the public health system has expanded to include adherence check-ins and counseling [29] and a variety of other key duties [30].

### Measures

#### Burnout

The MBI-HSS is considered the gold standard psychometric tool for measuring burnout in an occupational setting [31] and has previously been used in the healthcare setting in both South Africa [20] and Zambia [14]. The MBI-HSS comprises 22 items in three separate domains: emotional exhaustion, depersonalization, and personal accomplishment [32]. Each item is a statement, such as “I feel emotionally drained from my work”, and is scored based upon how often the respondent reports feeling this way about their job, with Likert responses from zero (“Never”) to six (“Every day”). Emotional exhaustion is defined as “feelings of being emotionally overextended and exhausted by one’s work”, and is measured using nine items, such as “I feel burned out from my work”, for a total score of zero to 54 [32]. Depersonalization is defined as “an unfeeling and impersonal response toward recipients of one’s service, care, treatment, or instruction” and is measured using five items, such as “I have become more callous toward people since I took this job”, for a total score of zero to 30 [32]. Personal accomplishment is defined as “feelings of competence and successful achievement in one’s work with people” and is measured using eight items, such as “I have accomplished many worthwhile things in this job”, for a total score of zero to 48 [32]. Burnout is defined as when emotional exhaustion and depersonalization are high and feelings of personal accomplishment are low [32].

Wording changes to the subscale items were made upon the recommendation of the local research staff in consultation with community advisory boards and the local country PIs. Ultimately, changes were made to three of the emotional exhaustion subscale items to improve respondents’ comprehension—the word “fatigued” was changed to “tired” in item 3, the phrase “a strain” was changed to “hard/difficult” in item 6, and the statement “I feel like I’m at the end of my rope” was changed to “I feel like I have reached the end” for item 20. In both countries, the MBI-HSS items were also translated into local languages (South Africa: Xhosa and Afrikaans; Zambia: Nyanja, Lozi, Tonga and Bemba). The accuracy of the translation was verified using back-translation.

The MBI-HSS has established cutoff values for categorizing respondents into “low”, “moderate”, and “high” categories of emotional exhaustion, depersonalization, and personal accomplishment. This study applied the cutoffs for the medical occupational subgroup provided in the MBI manual. Specifically, the cutoffs for emotional exhaustion were 0–18, 19–26 and 27–54; for depersonalization they were 0–33, 34–39 and 40–48; and for personal accomplishment they were 0–5, 6–9 and 10–30 for low, moderate, and high, respectively [32]

#### Sociodemographic characteristics

Sex was treated as a binary variable, with males as the reference group. Age was measured in years and categorized into groups aged 18–24, 25–34, 35–44, and older than 44, with those aged 18–24 as the reference group. Marital status was treated as a categorical variable with two levels: (1) not married (reference), including in a relationship but not living together, or separated, divorced or widowed, and (2) married or living together as if married. Level of education was treated as a categorical variable with three levels—less than secondary, completed secondary, or completed higher than secondary education. For the primary and stratified analyses, we combined the first two groups into one and used this group (i.e., completed secondary or below) as a reference.

#### Occupational characteristics

Length of time providing HIV services (in years) was treated as a continuous variable. To assess perceptions of the stigmatizing behaviors of their co-workers, health workers were asked a series of four, validated questions about whether they had witnessed their co-workers engaging in the following stigmatizing behaviors within the past 12 months: “My co-workers sometimes talk badly about people thought to be living with HIV”, “My co-workers sometimes gossip about clients’ HIV test results”, My co-workers sometimes treat people living with HIV poorly when providing them with health services”, and “My co-workers sometimes verbally insult clients living with HIV.” The original response options for these questions were “Strongly disagree”, “Disagree”, “Agree”, and “Strongly agree”. These were collapsed into a binary measure of those who “Did not agree or strongly agree with any of the four statements” vs “Agreed or strongly agreed with at least one of the four statements” [33]. Lastly, participants were asked whether they had ever been tested for HIV and, if they said yes, were asked to self-report, confidentially, the results of their latest HIV test. These self-reports were not confirmed through HIV testing.

### Statistical analyses

First, we explored the effect of missingness on analysis outcomes. Chi-squared tests for categorical variables and Wilcoxon rank sum tests for continuous variables were performed to determine whether significant differences existed between respondents with complete data on each subscale and those who were missing at least one item. The only factor that was consistently associated with item missingness across the three subscales was years providing HIV services; health workers who had provided services longer were less likely to be missing items within the burnout subscales. We concluded that the low level of missingness on each subscale (< 10%) and the general lack of significant differences between individuals who were or were not missing subscale items did not warrant imputation of missing values, and we chose to conduct complete case analysis for each scale (data not shown).

Proceeding with complete cases, next, confirmatory factor analysis was performed on the MBI-HSS data to check that the items loaded as expected onto 3 sub-scales. We also assessed reliability of the sub-scales by calculating Cronbach’s alpha. Alpha values equal to or greater than 0.70 were considered reliable for analysis. In Zambia, all the items loaded as expected, with Cronbach’s alpha values of 0.83 for emotional exhaustion, 0.80 for personal accomplishment, and 0.70 for depersonalization. In South Africa, three items were not strongly correlated with the other scale items on the subscale as expected: statement 4 from the personal accomplishment subscale (“I can easily understand how my clients feel about things”), statement 22 from the depersonalization subscale (“I feel clients blame me for some of their problems”), and statement 20 from the emotional exhaustion subscale (“I feel like I have reached the end”), which led to slightly lower alphas for two of the sub-scales. In South Africa, the value of Cronbach’s alpha for the scales was 0.84 for emotional exhaustion, 0.75 for personal accomplishment, and 0.65 for depersonalization. Despite the slight differences in the anticipated and actual correlations of some items in South Africa, we scored the MBI-HSS subscales as designed to allow comparison with other research studies.

Next, descriptive statistics for all risk factors and burnout subscales were calculated, and the distributions of the burnout scores were examined. The distributions of the depersonalization and personal accomplishment scores were heavily skewed. Specifically, more than four in five (81%) health workers scored “low” on the depersonalization subscale and more than half (57%) scored the minimum value of zero, while 81% of health workers scored “high” on the personal accomplishment subscale and 41% scored the maximum value of 48. Using emotional exhaustion as the central measure of burnout is supported by historical research cited in the MBI manual, which notes that “structural analyses contrasting models of burnout have generally found support for assigning a central, but not exclusive, role to emotional exhaustion. Exhaustion appears to be the MBI-HHS subscale that is most responsive to the organizational environment and social interactions that characterize human service work” [32]. Thus, we decided that only the emotional exhaustion subscale showed sufficient variation to warrant exploration of risk factors for this outcome.

Therefore, regression models for the emotional exhaustion subscale were created to test associations with six potential risk factors (i.e., variables associated with an increased risk of experiencing burnout, in this case): education, marital status, type of healthcare worker, number of years providing HIV services, HIV status, and witnessing stigmatizing behaviors of their co-workers. Following the recommendations provided in the MBI manual, the score on the emotional exhaustion subscale was treated as a continuous variable [32]. We fitted a linear regression model including a fixed term for community to account for the cluster-randomized trial design. Models were run twice—first, adjusting only for age, sex, and the study community (M1), and then again, additionally adjusting for marital status, level of education, type of health worker, and years providing HIV services (M2). We included the full sample (*n* = 1166) in models looking at the association between the sociodemographic and job-related characteristics. In models looking at the association between HIV status and emotional exhaustion (*n* = 1043), we excluded participants who reported they never tested, or refused to disclose the result of their last HIV test or had an undetermined result of their last HIV test. In models looking at the association between co-worker stigma and emotional exhaustion (*n* = 1092), we excluded participants with missing data on stigma. Finally, we examined the interaction between the effects of HIV status and co-worker stigma on emotional exhaustion and undertook stratified analysis by HIV status. For this analysis, we included health workers with complete data on stigma and excluded health workers who reported never tested, refused to disclose the result of their last HIV test, or had an undetermined result in their last HIV test (*n* = 985). All statistical analyses were conducted using the Stata software package (version 14.0, StataCorp LP, College Station, Texas). Significance of observed associations was assessed using 95% confidence intervals (CI) and *p*-values < 0.05.

## Results

### Study participants and descriptive statistics

Table 1 presents descriptive statistics for all sociodemographic and risk factor variables considered in this paper for 1166 health workers participating in the study.

**Table 1:** Sociodemographic and occupational risk factors of 1166 health workers providing HIV services in Zambia and South Africa, 2014–2015

Characteristics	Zambia						South Africa					
	Health facility staff (*n* = 351)		CHiPs (*n* = 356)		Community health workers (*n* = 73)		Health facility staff (*n* = 127)		CHiPs (*n* = 173)		Community health workers (*n* = 86)	
	*n*	%	*n*	%	*n*	%	*n*	%	*n*	%	*n*	%
Sex												
Male	110	31.3	134	37.6	16	21.9	15	11.8	37	21.4	0	0.0
Female	241	68.7	222	62.4	57	78.1	112	88.2	136	78.6	86	100.0
Age (years)												
18–24	32	9.1	20	5.6	3	4.1	13	10.2	26	15.0	11	12.8
25–34	101	28.8	115	32.3	19	26.0	37	29.1	97	56.1	29	33.7
35–44	84	23.9	95	26.7	10	13.7	39	30.7	38	22.0	29	33.7
> 44	134	38.2	126	35.4	41	56.2	38	29.9	12	6.9	17	19.8
Education												
Did not complete secondary	24	6.8	9	2.5	19	26.0	1	0.8	0	0.0	6	7.0
Completed secondary	103	29.3	162	45.5	40	54.8	51	40.2	120	69.4	74	86.0
Further	224	63.8	185	52.0	14	19.2	75	59.1	53	30.6	6	7.0
Marital status												
Not married	138	39.3	154	43.3	31	42.5	64	50.4	128	74.0	44	51.2
Married	213	60.7	202	56.7	42	57.5	63	49.6	45	26.0	42	48.8
Result of last HIV test												
Negative	264	75.2	235	66.0	48	65.8	94	74.0	143	82.7	50	58.1
Positive	49	14.0	96	27.0	16	21.9	20	15.7	12	6.9	16	18.6
Undetermined	1	0.3	2	0.6	1	1.4	0	0.0	0	0.0	2	2.3
Never tested	9	2.6	5	1.4	2	2.7	1	0.8	3	1.7	4	4.7
Refused	28	8.0	18	5.1	6	8.2	12	9.4	15	8.7	14	16.3
Perceived co-workers stigmatizing behavior reported by health workers												
Did not agree or strongly agree with any of four statements^a^	167	47.6	164	46.1	33	45.2	73	57.5	116	67.1	56	65.1
Agreed or strongly agreed with at least one of four statements^a^	169	48.1	173	48.6	38	52.1	40	31.5	40	23.1	23	26.7
Missing	15	4.3	19	5.3	2	2.7	14	11.0	17	9.8	7	8.1
Years providing HIV services												
Median		5		5		5		4		1		3
Mean		7		7		6		5		1		3
SD		5.8		4.8		4.7		4.3		1.0		3.3
Range	[0,30]		[0,32]		[0,21]		[0,24]		[0,6]		[0,16]	

^a^ Four statements were: “My co-workers sometimes talk badly about people thought to be living with HIV”, “My co-workers sometimes gossip about clients’ HIV test results”, “My co-workers sometimes treat people living with HIV poorly when providing them with health services”, and “My co-workers sometimes verbally insult clients living with HIV”

Most health workers were women in both countries and all cadres, ranging from 62% of CHiPs in Zambia to 100% of CHWs in South Africa. On average, health workers in Zambia, particularly the CHiPs, were older than those in South Africa; the largest proportion of health workers in all cadres fell in the 44 and above age range in Zambia, while the largest proportion of health workers in South Africa were 25–44 years of age. Most HFS, in both countries, had completed education beyond the secondary level. CHiPs in Zambia were more educated that those in South Africa, possibly a result of being older and no requirement that CHiPs in South Africa have previous work experience in HIV care. The majority of CHWs in both countries had completed secondary education. Between 57 and 61% of health workers in Zambia were married, compared to 26 to 50% of health workers in South Africa. CHiPs in South Africa were the least likely to be married (Table 1).

Most health workers (90%) had been tested for HIV and were willing to share the results of their latest test. The percentage of health workers who reported that their last HIV test was positive ranged from 7% of CHiPs in South Africa to 27% of CHiPs in Zambia. About half (48 to 52%) of each cadre of health worker in Zambia reported witnessing stigmatizing behaviors towards people living with HIV among their co-workers, while in South Africa, the percentage of health workers that reported witnessing stigmatizing behaviors ranged from 23% of CHiPs to 32% of HFS.

The median years health workers had provided HIV services was five years for all health worker cadres in Zambia, while in South Africa, HFS had provided services for a median of four years, CHWs had provided services for a median of three years, and CHiPs had provided services for a median of 1 year.

### Prevalence of burnout

**Fig. 2:**
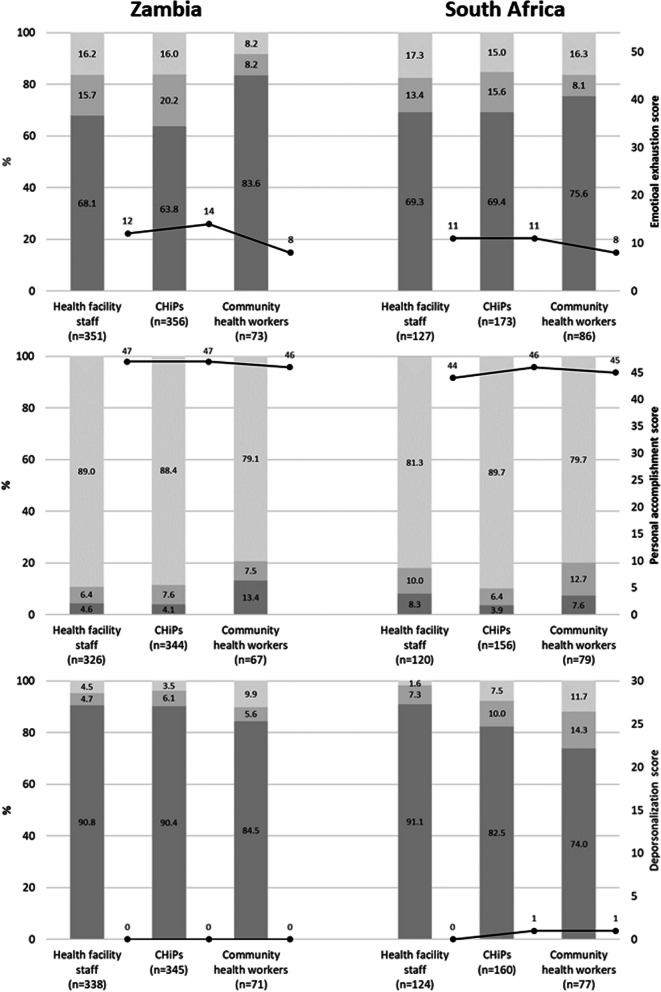
Median burnout subscale scores (dots with solid lines) and percentages of burnout subscale categorizations by different type of health worker providing HIV services in Zambia and South Africa, 2014–2015. Note: Sample sizes are 780 and 386 for the emotional exhaustion, 737 and 355 for personal accomplishment and 754 and 361 for depersonalization, for Zambia and South Africa, respectively. Burnout subscale categorization with dark grey for low and light grey for high burnout. Emotional exhaustion: low [0,18], Moderate [19, 26], high [27,54]; personal accomplishment: low [0,33], moderate [34, 39], high [40, 48]; depersonalization: low [0,5], moderate [6, 9], high [10, 30]

Tables 2 and 3 present health workers’ levels of emotional exhaustion, depersonalization, and personal accomplishment, which are also visualized in Fig. 2. On a scale from 0 to 54, the median emotional exhaustion was lowest, at 8, among CHWs in both countries, while median scores among HFS and CHiPs ranged from 11 to 14. Similarly, means were also lowest among CHWs (10.7 in Zambia and 12.0 in South Africa) and comparable among HFS and CHiPs in both countries (ranging from 14.3 to 15.4). There was little variation in the personal accomplishment score by country or health cadre; median scores ranged from 44 to 47 on a scale from zero to 48, while means ranged from 41.7 to 44.2. Similarly, there was little variation in depersonalization scores between health worker cadres in either country; the highest scores were reported by CHWs in South Africa, with a median score of one on a scale from zero to 30. Means across all cadres ranged from 2.1 to 4.6 (Table 2; Fig. 2).

**Table 2:** Burnout subscale scores of health workers providing HIV services in Zambia and South Africa, 2014–2015

Burnout subscale scores	Zambia			South Africa		
	Health facility staff	CHiPs	Community health workers	Health facility staff	CHiPs	Community health workers
Emotional exhaustion score [0,54], *n* = 1166						
*N*	351	356	73	127	173	86
Mean	14.7	15.4	10.7	14.4	14.3	12.0
Median	12	14	8	11	11	8
IQR	16	15	11	16	15	13
Range	[0,49]	[0,54]	[0,42]	[0,53]	[0,51]	[0,44]
Personal accomplishment score [0,48], *n* = 1092						
*N*	326	344	67	120	156	79
Mean	44.1	44.1	41.7	42.0	44.2	42.6
Median	47	47	46	44	46	45
IQR	6	6	7	7	6	7
Range	[0,48]	[1, 48]	[4, 48]	[7, 48]	[8, 48]	[17, 48]
Depersonalization score [0,30], *n* = 1115						
*N*	338	345	71	124	160	77
Mean	2.3	2.2	3.4	2.1	3.7	4.6
Median	0	0	0	0	1	1
IQR	3	2	5	4	6	7
Range	[0,30]	[0,30]	[0,30]	[0,18]	[0,28]	[0,27]

**Table 3:** Burnout subscale categorizations of health workers providing HIV services in Zambia and South Africa, 2014–2015

Burnout subscale categorization	Zambia						South Africa					
	Health facility staff		CHiPs		Community health workers		Health facility staff		CHiPs		Community health workers	
	*n*	%	*n*	%	*n*	%	*n*	%	*n*	%	*n*	%
Emotional exhaustion (*n* = 1166)												
Low [0,18]	239	68.1	227	63.8	61	83.6	88	69.3	120	69.4	65	75.6
Moderate [19, 26]	55	15.7	72	20.2	6	8.2	17	13.4	27	15.6	7	8.1
High [27,54]	57	16.2	57	16.0	6	8.2	22	17.3	26	15.0	14	16.3
Personal accomplishment (*n* = 1092)												
Low [0,33]	15	4.6	14	4.0	9	13.4	10	8.3	6	3.9	6	7.6
Moderate [34, 39]	21	6.4	26	7.6	5	7.5	12	10.0	10	6.4	10	12.7
High [40, 48]	290	89.0	304	88.4	53	79.1	120	81.7	140	89.7	63	79.8
Depersonalization score (*n* = 1115)												
Low [0,5]	307	90.8	312	90.4	60	84.5	113	91.1	132	82.5	57	74.0
Moderate [6, 9]	16	4.7	21	6.1	4	5.6	9	7.3	16	10.0	11	14.3
High [10, 30]	15	4.4	12	3.5	7	9.7	2	1.6	12	7.5	9	11.7

Between 68 and 83% of health workers in both countries and all cadres scored “low” on emotional exhaustion. Over 80% of health workers in both countries and all cadres scored “high” on personal accomplishment. Across health worker cadres, a higher percentage of CHWs in both countries scored “moderate” or “high” on depersonalization (15.3% in Zambia and 26.0% in South Africa). Still, most health workers in both countries and all cadres scored “low” on depersonalization, ranging from 82.5% among CHiPs in South Africa to 90.8% among HFS in Zambia (Table 3; Fig. 2). Applying the Maslach definition of burnout as when emotional exhaustion and depersonalization are high and feelings of personal accomplishment are low, just seven health workers (0.7%) would be scored as ‘burned out’.

### Risk factors for emotional exhaustion

In risk factor analysis adjusted for age, sex and community only (Model 1), completing further education beyond secondary school was associated with increased emotional exhaustion score compared to health workers who completed secondary education or below (*β* = 1.96, 95% CI 0.57 to 3.35). Marital status was not significantly associated with emotional exhaustion. Working as a CHW was significantly associated with lower emotional exhaustion compared to HFS (*β* = − 2.52, 95% CI − 4.69 to − 0.35). Emotional exhaustion among CHiPs was not significantly different from emotional exhaustion experienced by HFS. More years spent providing HIV services was significantly associated with increased emotional exhaustion (*β* = 0.22, 95% CI 0.06 to 0.38) (Table 4).

**Table 4:** The association between sociodemographic and occupational risk factors and emotional exhaustion (EE) among 1166 health workers providing HIV services in Zambia and South Africa, 2014–2015

Characteristics	Mean EE	M1		M2			
		*β* (95% CI)	*p*	*β*_adj_ (95% CI)^b^	*p*_adj_	*β*_adj_ (95% CI)^c^	*p*_adj_
Education^a^			0.006		0.110		0.003
Completed secondary or below (Ref.)	13.4	–		–		–	
Further	15.5	1.96 (0.57,3.35)		1.23 (− 0.28,2.76)		2.24 (0.76,3.72)	
Marital status			0.965		0.711		0.516
Not married (Ref.)	14.6	–		–		–	
Married	14.2	− 0.03 (− 1.47,1.40)		− 0.28 (− 1.77,1.21)		0.48 (− 0.97,1.94)	
Type of healthcare worker			0.003		0.003		0.003
Health facility staff (Ref.)	14.6	–		–		–	
CHiPs	15.1	1.07 (− 0.42,2.55)		1.74 (0.18,3.30)		1.71 (0.16,3.26)	
Community health worker	11.4	− 2.52 (− 4.69,− 0.35)		− 1.86 (− 4.26,0.55)		− 1.81 (− 4.11,0.49)	
Years providing HIV services	–	0.22 (0.06,0.38)	0.007	0.20 (0.03,0.36)	0.019	0.20 (0.03,0.36)	0.017
Result of last HIV test^b^			< 0.001		< 0.001		
Negative (Ref.)	15.1	–		–			
Positive	10.9	− 3.89 (− 5.66,− 2.11)		− 3.88 (− 5.69,− 2.07)			
Never tested, refused or undetermined	15.5	–		–			
Witnessed stigmatizing behaviors of their co-workers^c^	–		< 0.001				< 0.001
Disagree (Ref.)	12.8	–				–	
Agree	16.1	3.30 (1.90,4.69)				3.38 (1.99,4.76)	
Missing	16.2	–				–	

*β* regression coefficient; CI confidence interval; EE emotional exhaustion; *p* *p*-value of the Wald test; *β*_adj_ adjusted regression coefficient; *p*_adj_ adjusted *p*-value

M1 adjusted for age, sex and community; M2 fully confounder-adjusted model, adjusted for age, sex, education, marital status, type of healthcare worker, years providing HIV services and community

^a^Education was collapsed into two groups because of the small numbers in those not completing secondary education. The groups “Did not complete secondary” and “Completed secondary” were combined and used as the reference category

^b^Sample size for this model is *n* = 1043 due to respondents having never tested (*n* = 24), refusal to disclose result of last HIV test (*n* = 93), or an undetermined result of last HIV test (*n* = 6)

^c^Sample size for this model is *n* = 1092 due to missing data. Models include all health workers irrespective of HIV status

Controlling for age, sex, education, marital status, cadre of healthcare worker, years providing HIV services, and community (Model 2), health workers living with HIV had significantly lower emotional exhaustion (*β*adj = − 3.88, 95% CI − 5.69 to − 2.07) compared to health workers not living with HIV. In the fully adjusted model, CHiPs had significantly higher emotional exhaustion than HFS (*β*adj = 1.71, 95% CI 0.16 to 3.26). Among all health workers, irrespective of HIV status, witnessing stigmatizing behaviors towards people living with HIV among their co-workers was associated with significantly increased emotional exhaustion (*β*adj = 3.38, 95% CI 1.99 to 4.76) (Table 4). We found only weak evidence of an interaction between the effects of HIV status and co-worker stigma on emotional exhaustion (*p* = 0.11, Figure S1). In stratified analyses, health workers who self-reported as HIV-negative and witnessed stigmatizing behaviors towards people living with HIV among their co-workers had significantly higher emotional exhaustion (*β*adj = 3.40, 95% CI 1.74 to 5.06). We found no evidence of association between co-worker stigma and emotional exhaustion among health workers who self-reported HIV-positive (*β*adj = 0.99, 95% CI − 1.84 to 3.83) (Table S1).

## Discussion

In our study of 1166 health workers from three different cadres across South Africa and Zambia, less than 1% met Maslach’s criteria for occupational burnout at the beginning of the HPTN071 (PopART) trial. In contrast, over 80% of health workers reported low or moderate emotional exhaustion, over 90% reported low to moderate depersonalization, and over 85% reported moderate to high personal accomplishment. We identified several risk factors for emotional exhaustion among health workers, including higher levels of education, more years providing HIV services, testing negative for HIV at last test, being a community HIV care provider, and witnessing stigmatizing behavior towards people living with HIV among colleagues.

### Low levels of burnout

The level of burnout, as measured by the three sub-scales of the MBI-HSS, reported by our population of health workers is lower than both the reference values provided in the MBI manual [32] and previous studies using the MBI-HSS among similar populations [23, 34–36]. For example, the highest mean emotional exhaustion score among any cadre in any country was 15.4, compared to the mean of 18.3 reported among health workers providing HIV care in Malawi [35], and the mean of 22.2 reported in the manual for the medical occupation subgroup, which is based on an initial sample of 1104 American physicians and nurses [32]. All means are also lower than those found in a study conducted in 2003 among health workers in South Africa, which found a mean emotional exhaustion score of 24.2 [23]. Similarly, two unrelated studies in 2009 and 2018 conducted among health workers in Malawi found that more than one-third of health workers in the former scored high on the emotional exhaustion subscale while 55% of health workers in the latter scored moderate or high [34, 35]. A separate study conducted in South Africa also found that 35% of registered nurses experienced high emotional exhaustion [36]. For comparison, no more than 18% scored high on the emotional exhaustion subscale among any cadre in our study, and no more than 36% scored moderate or high.

There are several reasons why the reported levels of emotional exhaustion may have been lower in our study. One possible explanation is the timing of the study, which was conducted at the beginning of a large and well-known trial. On one hand, the start of the trial may have reduced levels of emotional exhaustion by giving providers new hope and tools to serve their clients living with HIV, including the ability to offer full ART access to all clients regardless of CD4 count in Arm A communities. On the other hand, the launch of the trial may have made health workers reticent to express feelings of burnout for fear of reprisal or, in the case of the CHiPs, loss of their new position.

Another possible explanation for lower than anticipated reported levels of emotional exhaustion could be problems with the translation or adaptation of the survey items. Every effort was made to reduce this possibility by completing a thorough back-translation process and consultation with indigenous language speakers in both countries. In Zambia, survey translations were available in Nyanja, Lozi, Tonga and Bemba in addition to English on the device. In South Africa, Xhosa and Afrikaans versions of the survey were available on the device in addition to the English version. However, most respondents chose to complete the survey in English. A preference to complete the survey in English may have led to differential interpretations of concepts and meaning by second language speakers. However, it is also possible that the use of self-administered surveys on the devices reduced levels of reporting bias. Finally, it is possible that health workers with higher levels of burnout were less likely to complete the survey at all, meaning that selection bias resulted in lower detected emotional exhaustion.

### Sociodemographic risk factors for emotional exhaustion

Similar to a study of burnout among VMMC providers in Kenya, most sociodemographic characteristics were not significantly associated with burnout [37]. Education level was the only significant sociodemographic risk factor for emotional exhaustion among the health workers in our study. Consistent with previous findings by Maslach et al. [24], we found that emotional exhaustion was significantly higher for those who completed higher education. Our findings are contrary, however, to those reported by Hu et al. in China, where higher levels of education were protective against emotional exhaustion for health workers [25], suggesting that influence of education may vary by context. The lack of significant difference by marital status observed in our study runs counter to findings reported among HIV care providers in Malawi [38], as well as by Maslach et al. [24]. While married people have previously been shown to experience lower levels of burnout, this effect may have been obscured in our study due to the large cohort of single CHiPs who were newly recruited into the study and had not previously provided HIV services.

### Occupational risk factors for emotional exhaustion

Our results suggest a positive association between years providing HIV services and emotional exhaustion among health workers in Zambia and South Africa. A previous systematic review in low- and middle-income countries, including studies from South Africa, Zambia and Uganda, found inconsistent results for the association between years providing HIV services and emotional exhaustion, with different studies showing both positive and negative associations [18]. Our results support the theory that the emotional care burden builds up among healthcare workers providing HIV services over time, which is consistent with evidence from Kenya among VMMC providers [37].

The cadre of health worker was also significantly associated with emotional exhaustion. Our findings that CHiPs reported the highest levels of emotional exhaustion, and CHWs reported the lowest levels, compared to HFS is interesting, as it suggests that work environment itself, facility-based versus community-based, may not alone determine levels emotional exhaustion. The low level of emotional exhaustion among CHWs may be partially due to differences in job attributes [18]. Specifically, CHWs were not trained as part of the PopART intervention and were therefore not beholden to meeting specific care targets related to the study implementation like the CHiPs were. Instead, they worked in less structured roles that allowed for some control over their work schedule, particularly in Zambia, which could have shielded CHWs from some of the targets or pressures to serve clients that other cadres faced. Lower emotional exhaustion among CHWs could also be reflective of their long-term presence in these communities, rooted in ideas around community responsibility, duty, and care, which may provide some protection from exhaustion [21]. At present, there are no other studies to compare our findings with about the work environment or attributes of specific community-based health worker roles. More research is needed to investigate the nuances surrounding community-based environments to ensure that health workers in this environment have access to the supports they need to cope with and protect against burnout.

Our finding that self-reported HIV-positive status was protective against emotional exhaustion is novel. Study staff familiar with health workers living with HIV in these settings shared that they had observed them taking particular care to ensure that their workload was manageable, which may have a protective effect against developing emotional exhaustion [39]. This suggests that future studies wishing to explore the relationship between HIV status and burnout among health workers should statistically adjust for their workload. Additionally, as discussed previously in relation to the CHWs, the motivation for employment in this line of work may also be protective for health care workers living with HIV, in that they have self-selected because of a deep sense of purpose and self-satisfaction derived from improving the health and wellbeing of others living with HIV, which may serve as a buffer against emotional exhaustion. Finally, because HIV status was self-reported, it is possible that health workers experiencing higher levels of internalized stigma and emotional exhaustion may have chosen not to disclose their HIV status.

The association between emotional exhaustion and witnessing a colleague stigmatize clients living with HIV indicates a relationship between observed stigma towards clients living with HIV in the health facility and the psychosocial wellbeing of health workers providing care, even though the health workers were not the target of the stigmatizing behaviors. This is corroborated by a study of South African nurses that examined various predictors of burnout using the MBI-HSS and observed a significant association between stigma and depersonalization [40]. This finding highlights the pernicious effect of stigma on the mental health and wellbeing of health workers [40], regardless of their HIV status, and demonstrates that stigma-reduction efforts in health care settings are crucial not only for the health and wellbeing of potential clients, but for the staff as well [41].

## Limitations

Our study had several strengths. We recruited a large and systematic, as opposed to opportunistic, sample of health workers from three cadres within 14 communities across two countries in sub-Saharan Africa. In addition, we used electronic capture devices that allowed participants to fill in responses to questions without the need to disclose their answers to an interviewer, reducing the likelihood of social desirability bias. However, a few limitations should be noted. Although the MBI-HSS has been used in SSA before, it was originally developed for higher income settings and the evidence of its psychometric properties in South Africa and Zambia is still emerging. To ensure that the MBI-HSS was understandable in our study context, we made a few minor changes to three items on the MBI-HSS, based on input from our Community Advisory Board, which may limit comparability of our findings with other studies. However, the CFA and reliability analyses for all but one sub-scale suggest strong psychometric properties in alignment with previous studies using the MBI-HSS in the region [36, 42]. The reliability of the depersonalization scale in South Africa was less than 0.7, so findings on this subscale should be interpreted with caution. Unlike other studies in the region, the depersonalization and personal accomplishment sub-scales were skewed towards the extremes, with little variability in responses. As such, we were not able to assess risk factors for either of these sub-scales in our analysis. As in many studies of health workers, response rates in our study were lower than in surveys in general populations, at 69.6% overall, but comparable to other surveys measuring HIV stigma and burnout among current and future health care workers with response rates in published literature as low as about 40% [43, 44]. In a previous baseline analysis, we noted that the most important reason for not being included in the survey was difficulty in locating respondents, as opposed to refusal to participate [27]. Lastly, we cannot exclude the possibility of residual confounding for factors that we were unable to measure in our study.

## Conclusion

Health workers participating in the rollout of this UTT intervention reported low levels of client depersonalization, high levels of personal accomplishment, and low levels of emotional exhaustion. The sociodemographic and occupational characteristics of health workers identified that may lead to greater emotional exhaustion can inform tailored interventions to protect the mental health and wellbeing of health workers and prevent burnout. For example, interventions to increase mindfulness [45, 46] and resilience [47] could be targeted towards more experienced health workers, those with higher levels of education, and those who are not living with HIV, to help them prevent burnout. Workplace wellness programs may also improve job satisfaction and reduce job stress and burnout [48]. In addition, whole-of-facility interventions to reduce HIV stigma in healthcare settings [49] may protect against emotional exhaustion across all cadres of health workers.

While we ultimately found that the HPTN071 (PopART) intervention did not significantly increase or decrease emotional exhaustion across the three arms at the end of the trial [50], it will be important for countries to monitor occupational burnout, including emotional exhaustion, among health workers as UTT is scaled up throughout the SSA region. Particularly in the context of emerging pandemics, like COVID-19, which place additional strain on already overburdened healthcare systems and health workforces in low- and middle-income countries [51–53]. Routine data on occupational burnout among the health workforce in SSA will be critical to ensure the longevity of the HIV service provider workforce in SSA, the provision of the highest quality services to people living with HIV, and pandemic preparedness strategies that will ensure a resilient and robust health workforce is in place to safeguard the public’s health.

### Supplementary Information

Supplementary Material 1.

## Data Availability

The datasets generated and/or analyzed during the current study are available in the ATLAS repository at SCHARP, https://atlas.scharp.org/cpas/project/home/begin.view?

## Abbreviations

ART: Antiretroviral therapy
CHiPs: Community HIV care providers
CHWs: Community health workers
HIV: Human Immunodeficiency Virus
MBI-HSS: Maslach Burnout Inventory-Human Services Survey
NGOs: Non-governmental organizations
SSA: Sub-Saharan Africa
UTT: Universal testing and treatment
