# Supplementary material for: Prevalence and risk of burnout among HIV service providers in South Africa and Zambia: findings from the HPTN 071 (PopART) trial
Source: Hum Resour Health. 2024 Jul 8;22:50. doi: 10.1186/s12960-024-00934-9 (PMC11229271; doi:10.1186/s12960-024-00934-9)
Supplement: Supplementary file 1 — Supplementary Material 1. [file 12960_2024_934_MOESM1_ESM.docx]

Table S1. Stratified analysis by HIV status looking at the association between sociodemographic and occupational risk factors and emotional exhaustion (EE) among 985 health workers ^a^ providing HIV services in Zambia and South Africa, 2014-2015.

| **Characteristics** | **HIV+ (n=200)** | | | | | | **HIV- (n=785)** | | | | | |
| --- | --- | --- | --- | --- | --- | --- | --- | --- | --- | --- | --- | --- |
|  |  | | **M1** | | **M2** | |  | | **M1** | | **M2** | |
|  | **N** | **Mean EE** | **β (95% CI)** | **p** | **β_adj_ (95% CI)** | **p_adj_** | **N** | **Mean EE** | **β (95% CI)** | **p** | **β_adj_ (95% CI)** | **p_adj_** |
| **Education ^b^** |  |  |  | 0.752 |  | 0.728 |  |  |  | **0.012** |  | 0.057 |
| Completed secondary or below (Ref.) | 145 | 10.5 | **-** |  | **-** |  | 369 | 14.0 | **-** |  | **-** |  |
| Further | 55 | 10.9 | 0.53 (-2.78,3.84) |  | 0.59 (-2.77,3.95) |  | 416 | 16.0 | 2.17 (0.47,3.87) |  | 1.73 (-0.05,3.50) |  |
| **Marital status** |  |  |  | 0.261 |  | 0.188 |  |  |  | 0.917 |  | 0.556 |
| Not married (Ref.) | 100 | 11.2 | **-** |  | - |  | 360 | 15.2 | - |  | - |  |
| Married | 100 | 10.0 | -1.72 (-4.73,1.29) |  | -2.06 (-5.13,1.02) |  | 425 | 15.0 | 0.09 (-1.68,1.87) |  | 0.53 (-1.23,2.29) |  |
| **Type of healthcare worker** |  |  |  | 0.447 |  | 0.336 |  |  |  | **<0.001** |  | **<0.001** |
| Health facility staff (Ref.) | 65 | 9.3 | - |  | - |  | 340 | 15.6 | - |  | - |  |
| CHiPs | 104 | 11.4 | 2.12 (-1.31,5.55) |  | 2.58 (-0.87,6.04) |  | 352 | 15.8 | 1.01 (-0.76,2.78) |  | 1.75 (-0.06,3.58) |  |
| Community health worker | 31 | 10.6 | 0.53 (-4.11,5.17) |  | 1.40 (-3.32,6.12) |  | 93 | 10.6 | -4.53 (-7.25,-1.80) |  | -3.25 (-6.10,-0.39) |  |
| **Years providing HIV services** |  | - | 0.27 (-0.08,0.62) |  | 0.31 (-0.05,0.66) | 0.088 |  | - | 0.14 (-0.05,0.34) | 0.145 | 0.14 (-0.05,0.33) | 0.153 |
| **Witnessed stigmatizing behaviors of their co-workers** |  |  |  | 0.405 |  | 0.491 |  |  |  |  |  |  |
| Disagree (Ref.) | 99 | 10.2 | - |  | - |  | 457 | 13.6 | - | **<0.001** | - | **<0.001** |
| Agree | 101 | 11.0 | 1.20 (-1.63,4.03) |  | 0.99 (-1.84,3.83) |  | 328 | 17.1 | 3.43 (1.76,5.11) |  | 3.40 (1.74,5.06) |  |

β regression coefficient; CI confidence interval; EE emotional exhaustion; p p-value of the Wald test; β**_adj_** adjusted regression coefficient; p**_adj_** adjusted p-value

M1 Adjusted for age, sex and community; M2 Fully confounder-adjusted model, adjusted for age, sex, education, marital status, type of healthcare worker, years providing HIV services and community.

^a^ Health workers who self-reported HIV-positive or HIV-negative with complete data on the four stigma statements.

^b^ Education was collapsed into two groups because of the small numbers in those not completing secondary education. The groups “Did not complete secondary” and “Completed secondary” were combined and used as the reference category.


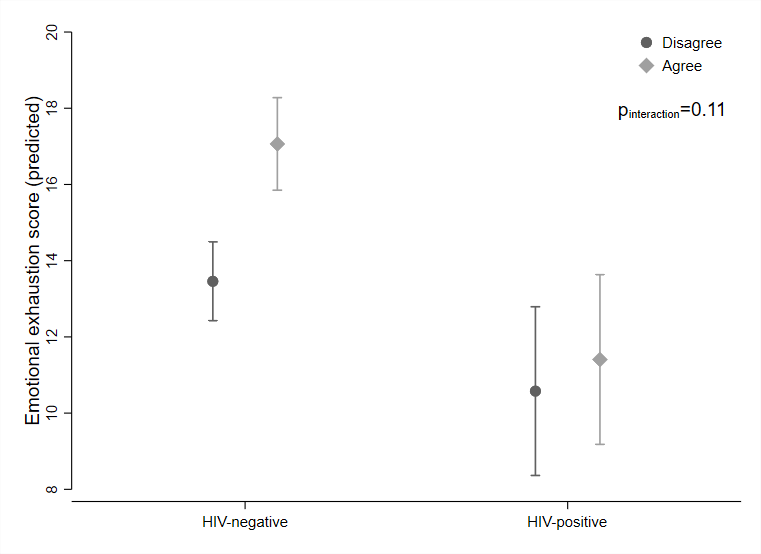


**Disagree**: Did not agree or strongly agree with any of four statements

**Agree**: Agreed or strongly agreed with at least one of four statements

Four statements were: “My co-workers sometimes talk badly about people thought to be living with HIV,” “My co-workers sometimes gossip about clients’ HIV test results,” “My co-workers sometimes treat people living with HIV poorly when providing them with health services,” and “My co-workers sometimes verbally insult clients living with HIV.”

Figure S1. Interaction between effects of co-worker stigma and self-reported HIV status on emotional exhaustion among 985 health workers.
